# Crystal structure and Hirshfeld surface analysis of the hydro­chloride salt of 8-{4-[(6-phenyl­pyridin-3-yl)meth­yl]piperazin-1-yl}-3,4-di­hydro­quinolin-2(1*H*)-one

**DOI:** 10.1107/S2056989021000979

**Published:** 2021-01-29

**Authors:** Nisar Ullah, Helen Stoeckli-Evans

**Affiliations:** a Department of Chemistry, King Fahd University of Petroleum and Minerals, 31261 Dahran, Saudi Arabia; b Institute of Physics, University of Neuchâtel, rue Emile-Argand 11, CH-2000 Neuchâtel, Switzerland

**Keywords:** crystal structure, di­hydro­quinolin-2(1*H*)-one, piperazine, hydro­chloride, mol­ecular salt, dopamine D_2_ receptor, serotonin 5-HT_1a_ receptor, hydrogen bonding, Hirshfeld surface analysis

## Abstract

The amine 8-{4-[(6-phenyl­pyridin-3-yl)meth­yl]piperazin-1-yl}-3,4-di­hydro­quinolin-2(1*H*)-one was crystallized as the hydro­chloride salt, 4-(2-oxo-1,2,3,4-tetra­hydro­quinolin-8-yl)-1-[(6-phenyl­pyridin-3-yl)meth­yl]piperazin-1-ium chloride, C_25_H_27_N_4_^+^·Cl^−^ (**I·HCl**). The conformation of the organic cation is half-moon in shape enclosing the chloride anion. The piperidine ring of the 3,4-di­hydro­quinolin-2(1*H*)-one moiety has a screw-boat conformation, while the piperazine ring has a chair conformation. In the biaryl group, the pyridine ring is inclined to the phenyl ring by 40.17 (7) and by 36.86 (8)° to the aromatic ring of the quinoline moiety. In the crystal, the cations are linked by pairwise N—H⋯O hydrogen bonds, forming inversion dimers enclosing an *R* ^2^_2_(8) ring motif. The Cl^−^ anion is linked to the cation by an N—H⋯Cl hydrogen bond. These units are linked by a series of C—H⋯O, C—H⋯N and C—H⋯Cl hydrogen bonds, forming layers lying parallel to the *ab* plane.

## Abstract 2

The amine 8-{4-[(6-phenyl­pyridin-3-yl)meth­yl]piperazin-1-yl}-3,4-di­hydro­quinolin-2(1*H*)-one was crystallized as the hydro­chloride salt 4-(2-oxo-1,2,3,4-tetra­hydro­quinolin-8-yl)-1-[(6-phenyl­pyridin-3-yl)meth­yl]piperazin-1-ium chloride. Its structure is compared to that of the salt 4-(2-oxo-1,2,3,4-tetra­hydro­quinolin-8-yl)-1-{[6-(4-fluoro­phen­yl)pyridin-3-yl]meth­yl}piperazin-1-ium chloride monohydrate, a fluorinated analogue.

## Chemical context   

**Figure 1:**
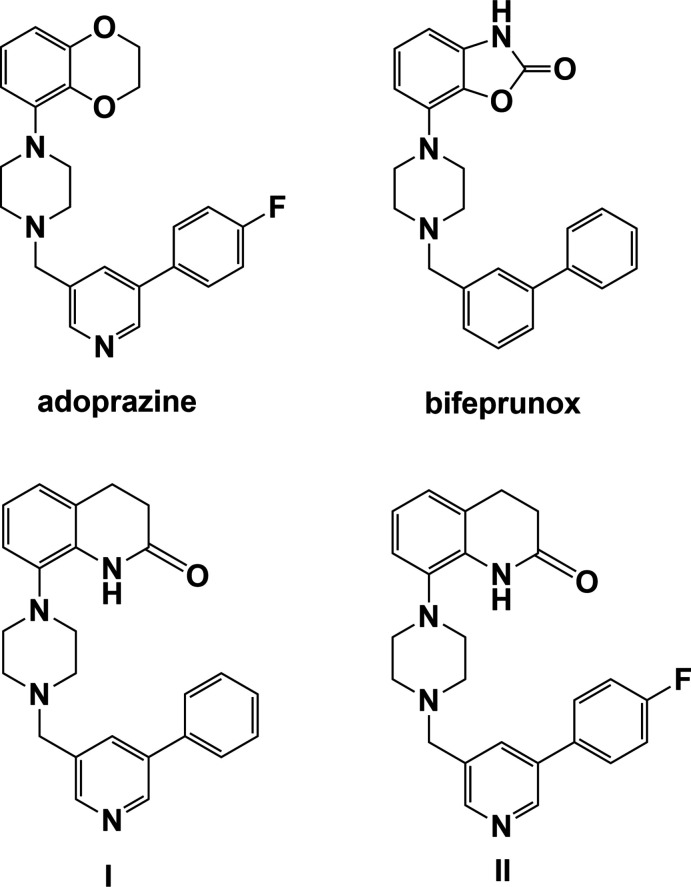
Chemical diagrams for adoprazine, bifeprunoc and compounds **I** and **II.**

Schizophrenia is a psychiatric illness afflicting over 1% of the world’s population. Adoprazine^©^ and Bifeprunox^©^ (Fig. 1 ▸) are two drugs that were developed for the treatment of Schizophrenia in the early 2000s. The main action of these two compounds is to combine dopamine D_2_ receptor blockade with serotonin 5-HT_1A_ receptor activation rather than antagonism (Feenstra *et al.*, 2001 ▸, 2006 ▸). In continuing efforts in this field, Ullah and collaborators have synthesized a series of compounds that are structural analogues of Adoprazine^©^ and Bifeprunox^©^ (Ullah, 2012 ▸, 2014*a* ▸,*b* ▸; Ullah & Al-Shaheri, 2012 ▸). These include a number of 1-aryl-4-(bi­aryl­methyl­ene)piperazines (Ullah, 2012 ▸), such as 8-{4-[(6-phenylpyridin-3-yl)methyl]piperazin-1-yl}-3,4-dihydroquinolin-2(1*H*)-one (**I**), and 8-(4-{[6-(4-fluorophenyl)pyridin-3-yl]methyl}piperazin-1-yl)-3,4-dihydroquinolin-2(1*H*)-one (**II**) Ghani *et al.* (2014 ▸) have reported that the D_2_ receptor binding affinity of compounds **I** and **II** are *K*_i_ = 28.4 n*M* for **I** and 42.0 n*M* for **II**. The 5-HT_1A_ receptor binding affinities were reported to be *K*_i_ = 4.30 n*M* for **I** and 52.5 n*M* for **II**. Hence, inserting a fluorine atom in the phenyl­pyridine unit in **II** did not improve its binding affinity compared to that of **I**. Full details concerning these assays are given in Ghani *et al.* (2014 ▸).

The crystal structure of the hydro­chloride salt of **II** has been reported previously (Ullah & Altaf, 2014 ▸) and will be compared here to that of the hydro­chloride salt of compound **I**.

## Structural commentary   

Due to the difficulty of forming suitable crystals for X-ray diffraction analysis compounds **I** and **II** were converted to their hydro­chloride salts by treatment with HCl in MeOH.
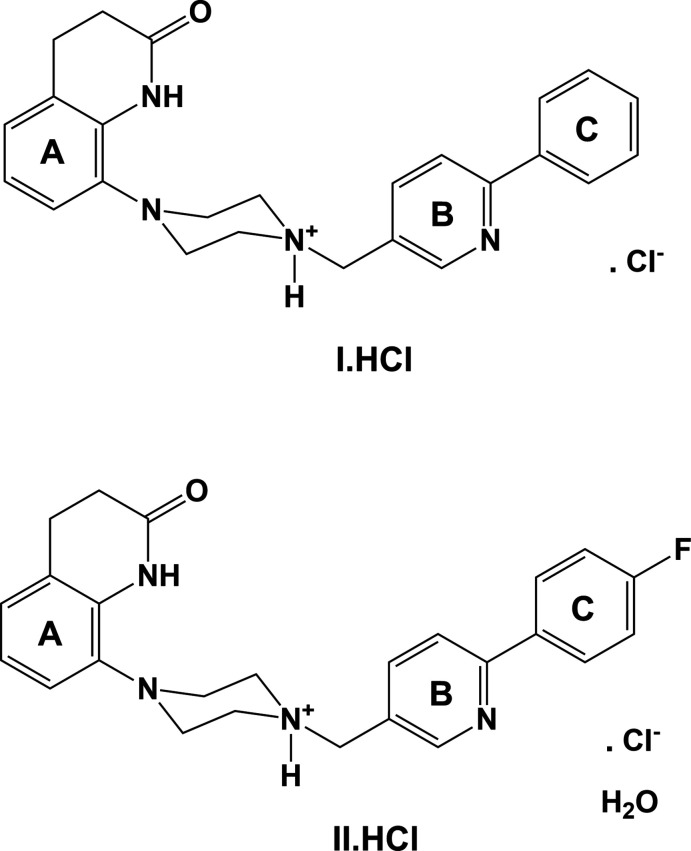


**Figure 2:**
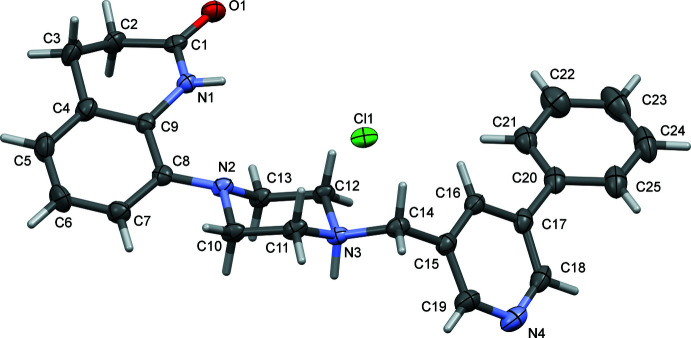
A view of the mol­ecular structure of **I·HCl**, with atom labelling. The displacement ellipsoids are drawn at the 50% probability level.

**Figure 3:**
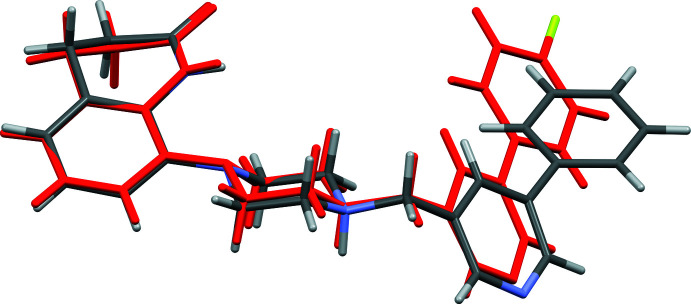
A view of the structural overlap of the cations of salts **I·HCl** and **II·HCl**; r.m.s. deviation 0.125 Å (*Mercury*; Macrae *et al.*, 2020 ▸). The structure of the **II·HCl** cation is given in red with the F atom in yellow (see also supplementary figure S1; Ullah & Altaf, 2014 ▸).

The organic cation of **I·HCl** has a half-moon shape enclosing the chloride anion (Fig. 2 ▸). The mol­ecular salt **II·HCl** crystallized as a monohydrate and here, while the cation also has a half-moon shape, it encloses the water mol­ecule of crystallization (Ullah & Altaf, 2014 ▸; see Fig. S1 in the supporting information). The two cations differ essentially in the conformation of the biaryl group (rings *B* = N4/C15–C19 and *C* = C20–C25) and their orientation with respect to the aromatic ring (*A* = C4–C9) of the 3,4-di­hydro­quinolin-2(1*H*)-one moiety. This is illustrated by the view of their structural overlap, shown in Fig. 3 ▸. In **I·HCl**, pyridine ring *B* is inclined to phenyl ring *C* by 40.17 (8)° while in **II·HCl** the equivalent dihedral angle is 10.06 (11)°. In **I·HCl**, ring *A* is inclined to rings *B* and *C* by 36.86 (8) and 14.16 (8)°, respectively. These dihedral angles differ considerably from the dihedral angles in **II·HCl**, where ring *A* is inclined to rings *B* and *C* by 51.20 (9) and 41.40 (11)°, respectively. In both compounds, the piperidine ring (N1/C1–C4/C9) has a screw-boat conformation with the torsion angle C1—C2—C3—C4 being −56.17 (18)° in **I·HCl** and −55.6 (2)° in **II·HCl**. In both compounds, the piperazine ring (N2/N3/C10–C13) has a chair conformation.

## Supra­molecular features   

**Figure 4:**
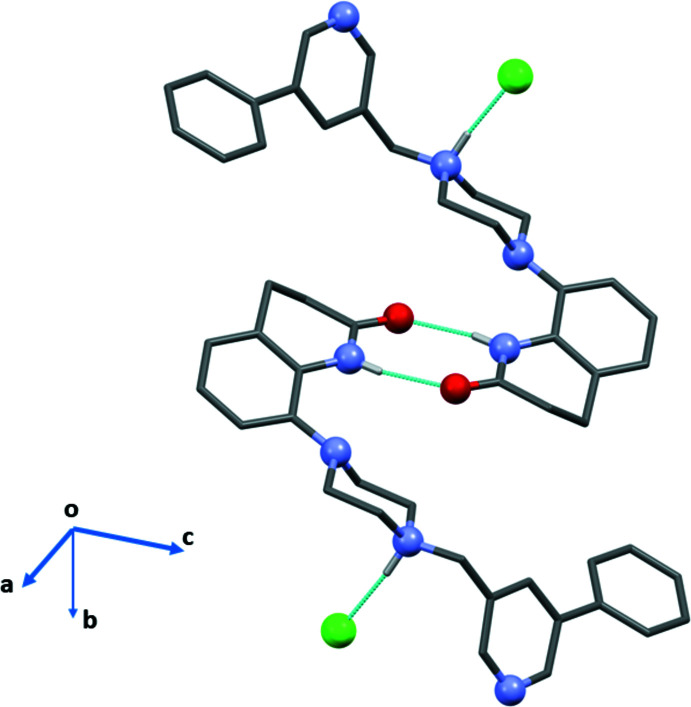
A view of the hydrogen bonded dimer formation in the crystal of salt **I·HCl**. Hydrogen bonds are shown as dashed lines (see Table 1 ▸).

**Figure 5:**
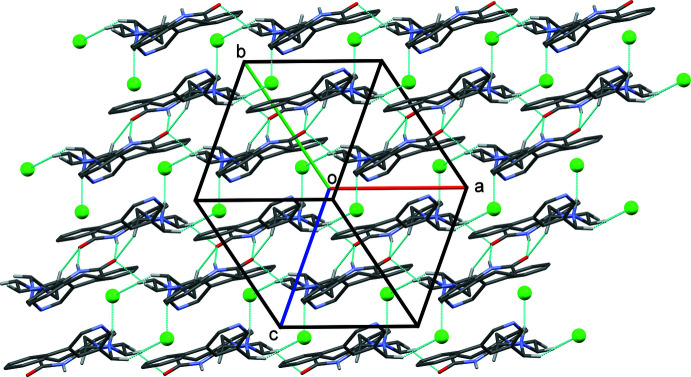
A view along the [111] direction of the crystal packing of salt **I·HCl**. Hydrogen bonds are shown as dashed lines (see Table 1 ▸).

In the crystal of **I·HCl**, the organic cations are linked by a pair of N—H⋯O hydrogen bonds, forming an inversion dimer enclosing an 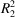
(8) ring motif (Fig. 4 ▸ and Table 1 ▸). The Cl^−^ anion is linked to the cation by an N—H⋯Cl hydrogen bond (Fig. 4 ▸ and Table 1 ▸). The dimers are linked by a C—H⋯O hydrogen bond, forming ribbons propagating along the *a*-axis direction. The ribbons are then linked *via* C—H⋯Cl hydrogen bonds to form layers lying parallel to the *ab* plane (Fig. 5 ▸ and Table 1 ▸). There are C—H⋯π(C4–C9) contacts present within the layers (Table 1 ▸), but there are no significant contacts present between the layers.

In the crystal of **II·HCl** (Ullah & Altaf, 2014 ▸; see Figs. S2 and S3, and Table S1 in the supporting information), the cations are linked by the water mol­ecules of crystallization *via* N—H⋯O_w_ and O_w_—H⋯O hydrogen bonds to form dimers with 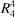
(12) ring motifs. The dimers are in turn linked by the Cl^−^ anions, *via* O_w_—H⋯Cl⋯H—N hydrogen bonds, to form chains propagating along the *b*-axis direction. The chains are linked *via* C—H⋯Cl and C—H⋯O hydrogen bonds, forming layers parallel to the *ab* plane.

In both cases, hydrogen-bonded layers are formed stacking along the *c-*axis direction and lying parallel to the *ab* plane. There are no significant directional inter-layer contacts present in either crystal structure.

## Hirshfeld surface analysis and two-dimensional fingerprint plots   

The Hirshfeld surface analysis (Spackman & Jayatilaka, 2009 ▸) and the associated two-dimensional fingerprint plots (McKinnon *et al.*, 2007 ▸) were performed with *CrystalExplorer17* (Turner *et al.*, 2017 ▸) following the protocol of Tiekink and collaborators (Tan *et al.*, 2019 ▸).

**Figure 6:**
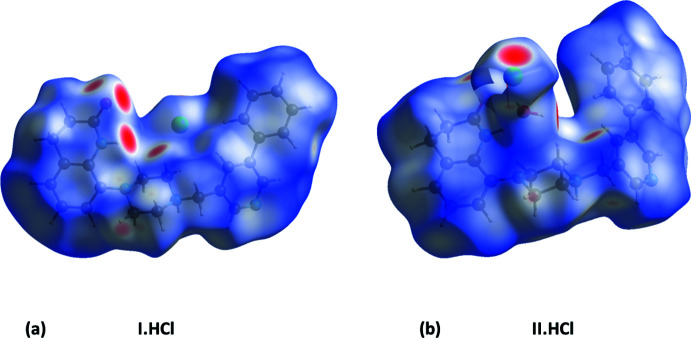
The Hirshfeld surfaces of compounds **I·HCl** and **II·HCl**, mapped over *d*_norm_ in the colour ranges of −0.5847 to 1.5642 au. and −0.5555 to 1.5111 au., respectively.

The Hirshfeld surfaces are colour-mapped with the normalized contact distance, *d*_norm_, varying from red (distances shorter than the sum of the van der Waals radii) through white to blue (distances longer than the sum of the van der Waals radii). The Hirshfeld surfaces (HS) of **I·HCl** and **II·HCl** mapped over *d*_norm_ are given in Fig. 6 ▸. It is evident from Fig. 6 ▸ *a* and 6*b* that there are important contacts present in the crystals of both compounds, the strong hydrogen bonds (Table 1 ▸ and Table S1) being indicated by the large red zones.

**Figure 7:**
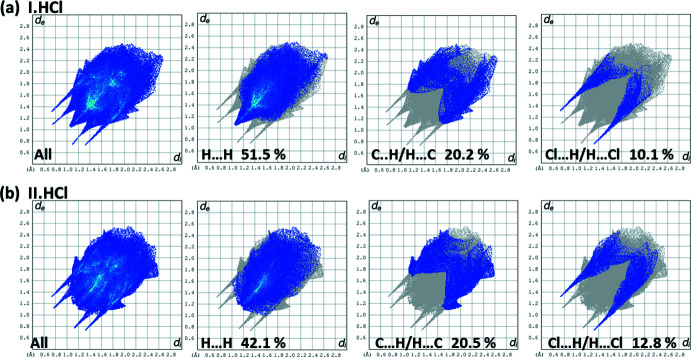
The two-dimensional fingerprint plots for compounds (*a*) **I·HCl** and (*b*) **II·HCl**, and those delineated into H⋯H, C⋯H/H⋯C, and Cl⋯H/H⋯Cl contacts.

The percentage contributions of inter-atomic contacts to the HS for both compounds are compared in Table 2 ▸. The two-dimensional fingerprint plots for the title salt, **I·HCl**, and those for **II·HCl**, are compared in Figs. 7 ▸ and 8 ▸. They reveal, as expected, that the principal contributions to the overall HS surface involve H⋯H contacts at 51.5 and 42.1%, respectively. The difference is attributed to the presence of F⋯H/H⋯F contacts in the crystal of **II·HCl**, amounting to 7.5%. The second most important contribution to the HS is from the C⋯H/H⋯C contacts at 20.2 and 20.5%, for **I·HCl** and **II·HCl**, respectively. These are followed by the Cl⋯H⋯Cl contacts at 10.1 and 12.8% for **I·HCl** and **II·HCl**, respectively, and O⋯H/H⋯O contacts at, respectively, 7.4 and 8.7%. The N⋯H/H⋯N contacts contribute, respectively, 6.5 and 5.3%. The C⋯C contacts in **I·HCl** contribute 2.9%, while the C⋯F contacts in **II·HCl** contribute 1.4%. All other atom⋯atom contacts contribute <1% to the HS for both compounds.

## Database survey   

**Figure 9:**
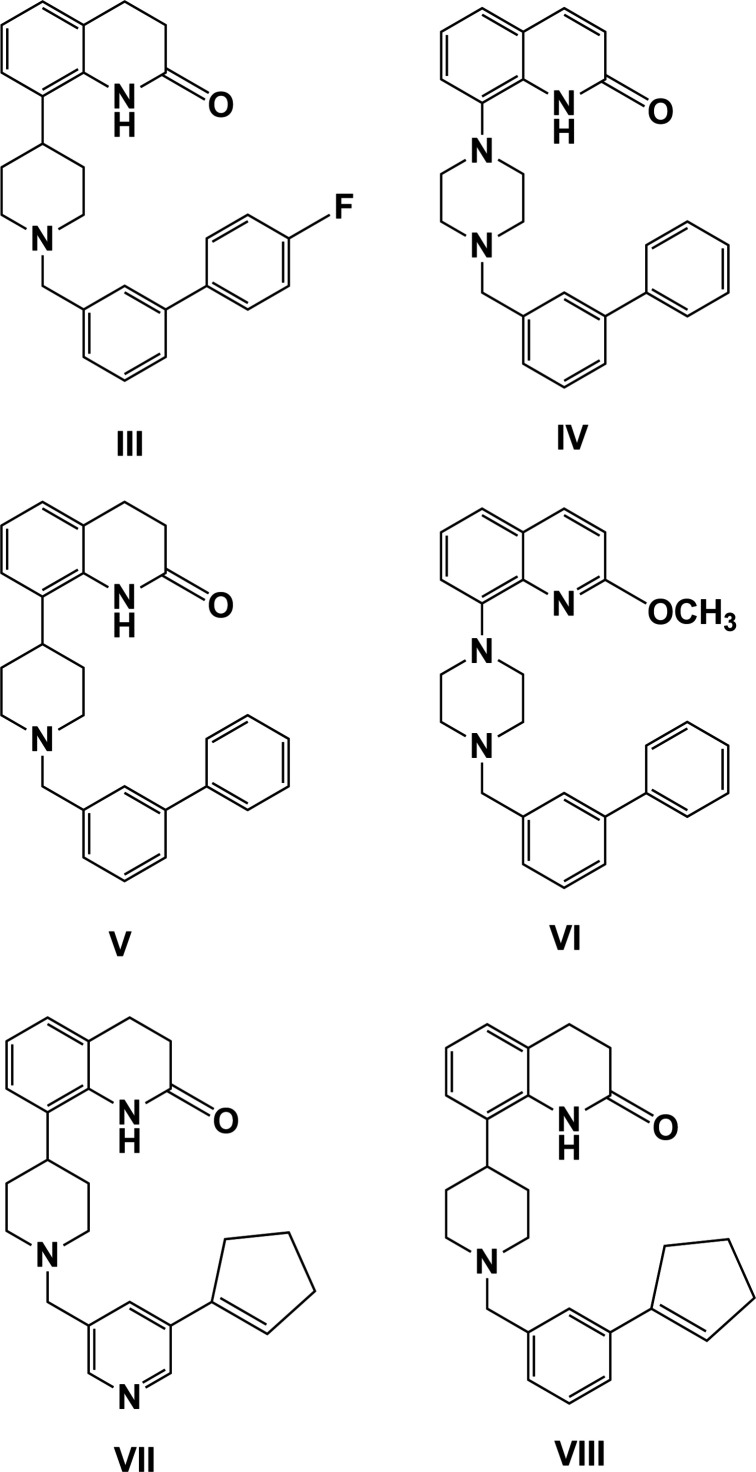
Chemical diagrams of similar compounds deposited with the CSD (Groom *et al.*, 2016 ▸); see § *5 Database survey.*

A search of the Cambridge Structural Database (CSD, Version 5.42, last update November 2020; Groom *et al.*, 2016 ▸) for 8-(piperazin-1-yl)-3,4-di­hydro­quinolin-2(1*H*)-ones gave three hits for compounds 8-{1-[(4′-fluoro-[1,1′-biphen­yl]-3-yl)meth­yl]piperidin-4-yl}-3,4-di­hydro­quinolin-2(1*H*)-one (**III**), that crystallized as a chloro­form 0.25-solvate (CSD refcode FITSEI; Ullah & Stoeckli-Evans, 2014 ▸), 8-[4-([1,1′-biphen­yl]-3-ylmeth­yl)piperazin-1-yl]quinolin-2(1*H*)-one (**IV**) (REYHIP; Ullah *et al.*, 2017 ▸) and 8-[1-([1,1′-biphen­yl]-3-ylmeth­yl)piperidin-4-yl]-3,4-di­hydro­quinolin-2(1*H*)-one (**V**) (REYHEL; Ullah *et al.*, 2017 ▸). Their chemical structures are shown in Fig. 9 ▸, together with those for compounds 8-[4-([1,1′-biphen­yl]-3-ylmeth­yl)piperazin-1-yl]-2-meth­oxy­quinoline (**VI**) (AKUXIQ; Ullah & Altaf, 2014 ▸), 8-(1-{[5-(cyclo­pent-1-en-1-yl)pyridin-3-yl]meth­yl}piperidin-4-yl)-3,4-di­hydro­quin­olin-2(1*H*)-one (**VII**) (AKUWOV; Ullah *et al.*, 2015 ▸) and 8-{1-[3-(cyclo­pent-1-en-1-yl)benz­yl]piperidin-4-yl}-3,4-di­hydro­quinolin-2(1*H*)-one (**VIII**) (AKUWUB; Ullah *et al.*, 2015 ▸). The CIFs for compounds **II·HCl** (AKUXEM; Ullah & Altaf, 2014 ▸) and **VI**–**VIII** have been updated recently in the CSD.

Compounds **III** to **VIII** all have a similar conformation; a curved or half-moon shape, as shown for the cation of **I·HCl** in Fig. 2 ▸. The piperidine rings of the di­hydro­quinoline units in compounds **I·HCl**, **II·HCl**, **III**, **V**, **VII** and **VIII** have screw-boat conformations. The piperidine or piperazine rings in all eight compounds have chair conformations.

## Synthesis and crystallization   

The synthesis of compounds **I** and **II** has been reported (Ullah, 2012 ▸; compounds 5*c* and 5*d* in that paper). Colourless plate-like crystals of their hydro­chloride salts were obtained by slow evaporation of solutions in di­chloro­methane and methanol; ratios (8:3) and (8.5:1.5), respectively.

## Refinement   

Crystal data, data collection and structure refinement details are summarized in Table 3 ▸. The NH H atoms were located in a difference electron-density map and freely refined. The C-bound H atoms were included in calculated positions and refined as riding on the parent atom: C—H = 0.95–0.99 Å with *U*_iso_(H) = 1.2*U*_eq_(C).

## Supplementary Material

Crystal structure: contains datablock(s) I, Global. DOI: 10.1107/S2056989021000979/hb7965sup1.cif

Structure factors: contains datablock(s) I. DOI: 10.1107/S2056989021000979/hb7965Isup2.hkl

Details concerning structure II.HCl. DOI: 10.1107/S2056989021000979/hb7965sup3.pdf

CCDC reference: 2059142

Additional supporting information:  crystallographic information; 3D view; checkCIF report

## Figures and Tables

**Figure 8:**
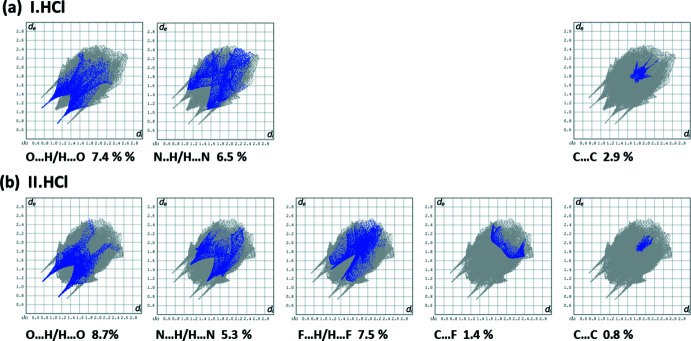
The two-dimensional fingerprint plots for compounds (*a*) **I·HCl** delineated into O⋯H/H⋯O, N⋯H/H⋯N and C⋯C, and (*b*) **II·HCl** delineated into O⋯H/H⋯O, N⋯H/H⋯N, F⋯H/H⋯F, C⋯F/F⋯C and C⋯C contacts.

**Table 1:** Hydrogen-bond geometry (Å, °) *Cg* is the centroid of the C4–C9 ring.

*D*—H⋯*A*	*D*—H	H⋯*A*	*D*⋯*A*	*D*—H⋯*A*
N1—H1*N*⋯O1^i^	0.85 (2)	2.01 (2)	2.844 (2)	168 (2)
N3—H3*N*⋯Cl1^ii^	0.97 (2)	2.12 (2)	3.065 (1)	167 (2)
C10—H10*B*⋯O1^iii^	0.99	2.40	3.151 (2)	132
C11—H11*A*⋯Cl1^iii^	0.99	2.80	3.668 (2)	147
C12—H12*A*⋯Cl1	0.99	2.81	3.520 (2)	129
C12—H12*B*⋯O1^i^	0.99	2.26	3.123 (2)	144
C13—H13*A*⋯N1	0.99	2.53	3.138 (2)	120
C14—H14*A*⋯Cl1^iii^	0.99	2.71	3.585 (2)	147
C21—H21⋯Cl1	0.95	2.83	3.757 (2)	165
C18—H18⋯*Cg* ^ii^	0.95	2.83	3.487 (2)	127

Symmetry codes: (i) 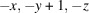; (ii) 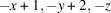; (iii) 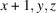.

**Table 2:** Principal percentage contributions of inter-atomic contacts to the Hirshfeld surfaces of **I·HCl** and **II·HCl**.

Contact	**I·HCl** % contribution	**II·HCl** % contribution
H⋯H	51.5	42.1
C⋯H/H⋯C	20.2	20.5
Cl⋯H/H⋯Cl	10.1	12.8
O⋯H/H⋯O	7.4	8.7
N⋯H/H⋯N	6.5	5.3
F⋯H/H⋯F	–	7.5
C⋯F/F⋯C	–	1.4
C⋯C	2.9	0.8

**Table 3:** Experimental details

Crystal data	
Chemical formula	C_25_H_27_N_4_O^+^·Cl^−^
*M*_r_	434.95
Crystal system, space group	Triclinic, *P* 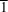
Temperature (K)	173
*a*, *b*, *c* (Å)	8.4791 (8), 10.4091 (10), 13.6862 (14)
α, β, γ (°)	90.138 (8), 94.833 (8), 113.745 (7)
*V* (Å^3^)	1100.88 (19)
*Z*	2
Radiation type	Mo *K*α
μ (mm^−1^)	0.20
Crystal size (mm)	0.45 × 0.33 × 0.18

Data collection	
Diffractometer	Stoe *IPDS* 2
Absorption correction	Multi-scan (*MULABS*; Spek, 2020 ▸)
*T*_min_, *T*_max_	0.379, 1.000
No. of measured, independent and observed [*I* > 2σ(*I*)] reflections	13410, 4156, 2912
*R*_int_	0.077
(sin θ/λ)_max_ (Å^−1^)	0.609

Refinement	
*R*[*F* ^2^ > 2σ(*F* ^2^)], *wR*(*F* ^2^), *S*	0.033, 0.071, 0.83
No. of reflections	4156
No. of parameters	289
H-atom treatment	H atoms treated by a mixture of independent and constrained refinement
Δρ_max_, Δρ_min_ (e Å^−3^)	0.22, −0.22

Computer programs: *X-AREA* (Stoe & Cie, 2009 ▸), *X-RED32* (Stoe & Cie, 2009 ▸), *SHELXS97* (Sheldrick, 2008 ▸), *Mercury* (Macrae *et al.*, 2020 ▸), *SHELXL2018/3* (Sheldrick, 2015 ▸), *PLATON* (Spek, 2020 ▸) and *publCIF* (Westrip, 2010 ▸).

## supplementary crystallographic information

### Crystal data

**Table:**

C_25_H_27_N_4_O^+^·Cl^−^	*Z* = 2
*M**_r_* = 434.95	*F*(000) = 460
Triclinic, *P*1	*D*_x_ = 1.312 Mg m^−^^3^
*a* = 8.4791 (8) Å	Mo *K*α radiation, λ = 0.71073 Å
*b* = 10.4091 (10) Å	Cell parameters from 9997 reflections
*c* = 13.6862 (14) Å	θ = 1.5–26.1°
α = 90.138 (8)°	µ = 0.20 mm^−^^1^
β = 94.833 (8)°	*T* = 173 K
γ = 113.745 (7)°	Rod, colourless
*V* = 1100.88 (19) Å^3^	0.45 × 0.33 × 0.18 mm

### Data collection

**Table:**

Stoe IPDS 2 diffractometer	4156 independent reflections
Radiation source: fine-focus sealed tube	2912 reflections with *I* > 2σ(*I*)
Plane graphite monochromator	*R*_int_ = 0.077
φ + ω scans	θ_max_ = 25.6°, θ_min_ = 1.5°
Absorption correction: multi-scan (MULABS; Spek, 2020)	*h* = −10→9
*T*_min_ = 0.379, *T*_max_ = 1.000	*k* = −12→12
13410 measured reflections	*l* = −16→16

### Refinement

**Table:**

Refinement on *F*^2^	Secondary atom site location: difference Fourier map
Least-squares matrix: full	Hydrogen site location: mixed
*R*[*F*^2^ > 2σ(*F*^2^)] = 0.033	H atoms treated by a mixture of independent and constrained refinement
*wR*(*F*^2^) = 0.071	*w* = 1/[σ^2^(*F*_o_^2^) + (0.0262*P*)^2^] where *P* = (*F*_o_^2^ + 2*F*_c_^2^)/3
*S* = 0.83	(Δ/σ)_max_ = 0.001
4156 reflections	Δρ_max_ = 0.22 e Å^−^^3^
289 parameters	Δρ_min_ = −0.22 e Å^−^^3^
0 restraints	Extinction correction: (SHELXL-2018/3; Sheldrick, 2015), Fc^*^=kFc[1+0.001xFc^2^λ^3^/sin(2θ)]^-1/4^
Primary atom site location: structure-invariant direct methods	Extinction coefficient: 0.0061 (11)

### Special details

**Table:**

Geometry. All esds (except the esd in the dihedral angle between two l.s. planes) are estimated using the full covariance matrix. The cell esds are taken into account individually in the estimation of esds in distances, angles and torsion angles; correlations between esds in cell parameters are only used when they are defined by crystal symmetry. An approximate (isotropic) treatment of cell esds is used for estimating esds involving l.s. planes.

### Fractional atomic coordinates and isotropic or equivalent isotropic displacement parameters (Å^2^)

**Table:**

	*x*	*y*	*z*	*U*_iso_*/*U*_eq_	
O1	−0.17052 (13)	0.47203 (11)	−0.07307 (8)	0.0288 (3)	
N1	0.07882 (16)	0.49233 (13)	−0.13301 (10)	0.0234 (3)	
H1N	0.122 (2)	0.5073 (18)	−0.0734 (13)	0.034 (5)*	
N2	0.44879 (14)	0.63611 (12)	−0.11540 (9)	0.0220 (3)	
N3	0.68062 (15)	0.82122 (13)	0.04180 (9)	0.0217 (3)	
H3N	0.746 (2)	0.906 (2)	0.0090 (13)	0.052 (5)*	
N4	0.94060 (18)	1.20937 (14)	0.25205 (11)	0.0399 (4)	
C1	−0.09235 (18)	0.46487 (15)	−0.14318 (11)	0.0236 (3)	
C2	−0.1799 (2)	0.42765 (18)	−0.24529 (12)	0.0329 (4)	
H2A	−0.160759	0.514512	−0.280987	0.040*	
H2B	−0.306002	0.375119	−0.242625	0.040*	
C3	−0.1077 (2)	0.33797 (19)	−0.29954 (13)	0.0392 (4)	
H3A	−0.135909	0.247286	−0.267359	0.047*	
H3B	−0.160866	0.318222	−0.368136	0.047*	
C4	0.0858 (2)	0.41520 (16)	−0.29844 (12)	0.0302 (4)	
C5	0.1751 (2)	0.41199 (19)	−0.37763 (13)	0.0392 (4)	
H5	0.114179	0.360241	−0.436140	0.047*	
C6	0.3535 (2)	0.48412 (19)	−0.37196 (13)	0.0392 (4)	
H6	0.414790	0.480812	−0.426264	0.047*	
C7	0.4424 (2)	0.56092 (17)	−0.28716 (12)	0.0310 (4)	
H7	0.564389	0.610806	−0.284514	0.037*	
C8	0.35719 (18)	0.56693 (15)	−0.20549 (11)	0.0230 (3)	
C9	0.17520 (18)	0.49231 (15)	−0.21240 (11)	0.0228 (3)	
C10	0.63546 (18)	0.67238 (16)	−0.10867 (11)	0.0266 (4)	
H10A	0.692624	0.755875	−0.147562	0.032*	
H10B	0.656720	0.593449	−0.136332	0.032*	
C11	0.71077 (19)	0.70267 (16)	−0.00407 (11)	0.0256 (4)	
H11A	0.836721	0.727272	−0.001024	0.031*	
H11B	0.657845	0.617257	0.033766	0.031*	
C12	0.49249 (17)	0.79433 (16)	0.02658 (11)	0.0238 (3)	
H12A	0.476973	0.878517	0.049257	0.029*	
H12B	0.425884	0.715170	0.066760	0.029*	
C13	0.42172 (18)	0.75986 (15)	−0.08004 (11)	0.0232 (3)	
H13A	0.296647	0.739395	−0.086798	0.028*	
H13B	0.481455	0.841377	−0.120110	0.028*	
C14	0.74694 (19)	0.83801 (16)	0.14807 (11)	0.0271 (4)	
H14A	0.863559	0.836905	0.153532	0.033*	
H14B	0.670376	0.755999	0.182549	0.033*	
C15	0.75825 (19)	0.96984 (16)	0.19971 (11)	0.0259 (4)	
C16	0.6286 (2)	0.97397 (16)	0.25369 (11)	0.0276 (4)	
H16	0.521948	0.894116	0.253316	0.033*	
C17	0.6549 (2)	1.09519 (16)	0.30836 (11)	0.0289 (4)	
C18	0.8145 (2)	1.20792 (17)	0.30499 (12)	0.0354 (4)	
H18	0.835305	1.290278	0.343377	0.042*	
C19	0.9109 (2)	1.09080 (17)	0.20072 (13)	0.0351 (4)	
H19	0.998875	1.089041	0.162807	0.042*	
C20	0.5209 (2)	1.10509 (17)	0.36734 (12)	0.0326 (4)	
C21	0.3467 (2)	1.04802 (18)	0.33182 (13)	0.0366 (4)	
H21	0.312714	1.001744	0.268836	0.044*	
C22	0.2225 (3)	1.0581 (2)	0.38747 (14)	0.0480 (5)	
H22	0.103978	1.017354	0.362924	0.058*	
C23	0.2710 (3)	1.1276 (2)	0.47900 (15)	0.0557 (6)	
H23	0.186196	1.135071	0.517087	0.067*	
C24	0.4438 (3)	1.1856 (2)	0.51400 (14)	0.0515 (5)	
H24	0.477753	1.233593	0.576400	0.062*	
C25	0.5676 (3)	1.17463 (19)	0.45928 (13)	0.0436 (5)	
H25	0.685829	1.214852	0.484499	0.052*	
Cl1	0.16918 (5)	0.91117 (4)	0.07387 (3)	0.02965 (12)	

### Atomic displacement parameters (Å^2^)

**Table:**

	*U*^11^	*U*^22^	*U*^33^	*U*^12^	*U*^13^	*U*^23^
O1	0.0214 (5)	0.0315 (6)	0.0325 (6)	0.0094 (5)	0.0034 (5)	0.0009 (5)
N1	0.0193 (6)	0.0251 (7)	0.0233 (8)	0.0067 (5)	0.0005 (6)	−0.0016 (6)
N2	0.0166 (6)	0.0211 (7)	0.0291 (7)	0.0082 (5)	0.0028 (5)	−0.0021 (5)
N3	0.0168 (6)	0.0190 (6)	0.0284 (7)	0.0063 (5)	0.0026 (5)	0.0015 (5)
N4	0.0338 (8)	0.0276 (8)	0.0486 (9)	0.0042 (6)	−0.0047 (7)	−0.0027 (7)
C1	0.0190 (7)	0.0168 (7)	0.0326 (9)	0.0045 (6)	0.0034 (7)	0.0017 (6)
C2	0.0223 (8)	0.0363 (10)	0.0332 (9)	0.0056 (7)	−0.0024 (7)	−0.0034 (8)
C3	0.0324 (9)	0.0379 (10)	0.0365 (10)	0.0046 (8)	−0.0038 (8)	−0.0151 (8)
C4	0.0314 (9)	0.0255 (9)	0.0318 (9)	0.0100 (7)	0.0002 (7)	−0.0055 (7)
C5	0.0440 (10)	0.0421 (11)	0.0304 (10)	0.0168 (8)	0.0004 (8)	−0.0131 (8)
C6	0.0448 (11)	0.0452 (11)	0.0306 (10)	0.0196 (9)	0.0129 (8)	−0.0062 (8)
C7	0.0277 (8)	0.0314 (9)	0.0346 (9)	0.0120 (7)	0.0074 (7)	−0.0004 (7)
C8	0.0253 (8)	0.0197 (8)	0.0259 (8)	0.0109 (6)	0.0035 (7)	0.0009 (6)
C9	0.0253 (8)	0.0182 (7)	0.0255 (8)	0.0089 (6)	0.0037 (7)	−0.0004 (6)
C10	0.0182 (7)	0.0260 (8)	0.0366 (9)	0.0093 (6)	0.0059 (7)	−0.0005 (7)
C11	0.0184 (7)	0.0223 (8)	0.0383 (10)	0.0105 (6)	0.0021 (7)	−0.0009 (7)
C12	0.0152 (7)	0.0248 (8)	0.0313 (9)	0.0079 (6)	0.0029 (6)	−0.0023 (6)
C13	0.0183 (7)	0.0217 (8)	0.0302 (9)	0.0091 (6)	0.0007 (6)	−0.0018 (6)
C14	0.0217 (8)	0.0273 (9)	0.0311 (9)	0.0092 (6)	−0.0006 (7)	0.0032 (7)
C15	0.0261 (8)	0.0246 (8)	0.0242 (8)	0.0085 (6)	−0.0037 (7)	0.0011 (6)
C16	0.0281 (8)	0.0232 (8)	0.0267 (9)	0.0063 (6)	−0.0019 (7)	0.0001 (7)
C17	0.0379 (9)	0.0271 (9)	0.0216 (9)	0.0144 (7)	−0.0041 (7)	−0.0001 (7)
C18	0.0448 (10)	0.0227 (9)	0.0325 (10)	0.0099 (7)	−0.0103 (8)	−0.0043 (7)
C19	0.0278 (9)	0.0305 (10)	0.0404 (10)	0.0057 (7)	−0.0010 (8)	−0.0003 (8)
C20	0.0492 (11)	0.0253 (9)	0.0252 (9)	0.0179 (8)	−0.0002 (8)	0.0013 (7)
C21	0.0497 (11)	0.0336 (10)	0.0296 (9)	0.0197 (8)	0.0049 (8)	0.0003 (7)
C22	0.0534 (12)	0.0526 (12)	0.0471 (12)	0.0299 (10)	0.0099 (10)	0.0062 (10)
C23	0.0860 (17)	0.0597 (14)	0.0428 (13)	0.0481 (13)	0.0242 (12)	0.0077 (10)
C24	0.0872 (17)	0.0492 (12)	0.0286 (11)	0.0380 (12)	0.0067 (11)	−0.0037 (9)
C25	0.0672 (13)	0.0374 (10)	0.0294 (10)	0.0259 (9)	−0.0023 (9)	−0.0025 (8)
Cl1	0.02283 (19)	0.0261 (2)	0.0381 (2)	0.00758 (15)	0.00489 (17)	0.00481 (17)

### Geometric parameters (Å, º)

**Table:**

O1—C1	1.2298 (17)	C10—H10B	0.9900
N1—C1	1.3562 (19)	C11—H11A	0.9900
N1—C9	1.4136 (19)	C11—H11B	0.9900
N1—H1N	0.852 (17)	C12—C13	1.515 (2)
N2—C8	1.4233 (19)	C12—H12A	0.9900
N2—C10	1.4665 (18)	C12—H12B	0.9900
N2—C13	1.4832 (19)	C13—H13A	0.9900
N3—C14	1.4981 (19)	C13—H13B	0.9900
N3—C12	1.5002 (17)	C14—C15	1.507 (2)
N3—C11	1.5031 (19)	C14—H14A	0.9900
N3—H3N	0.967 (18)	C14—H14B	0.9900
N4—C18	1.336 (2)	C15—C16	1.390 (2)
N4—C19	1.340 (2)	C15—C19	1.395 (2)
C1—C2	1.498 (2)	C16—C17	1.394 (2)
C2—C3	1.527 (2)	C16—H16	0.9500
C2—H2A	0.9900	C17—C18	1.394 (2)
C2—H2B	0.9900	C17—C20	1.484 (2)
C3—C4	1.507 (2)	C18—H18	0.9500
C3—H3A	0.9900	C19—H19	0.9500
C3—H3B	0.9900	C20—C25	1.395 (2)
C4—C5	1.381 (2)	C20—C21	1.393 (2)
C4—C9	1.400 (2)	C21—C22	1.387 (2)
C5—C6	1.387 (2)	C21—H21	0.9500
C5—H5	0.9500	C22—C23	1.389 (3)
C6—C7	1.384 (2)	C22—H22	0.9500
C6—H6	0.9500	C23—C24	1.382 (3)
C7—C8	1.395 (2)	C23—H23	0.9500
C7—H7	0.9500	C24—C25	1.380 (3)
C8—C9	1.415 (2)	C24—H24	0.9500
C10—C11	1.496 (2)	C25—H25	0.9500
C10—H10A	0.9900		
			
C1—N1—C9	123.74 (14)	C10—C11—H11B	109.3
C1—N1—H1N	112.8 (11)	N3—C11—H11B	109.3
C9—N1—H1N	123.5 (11)	H11A—C11—H11B	107.9
C8—N2—C10	114.51 (12)	N3—C12—C13	112.12 (12)
C8—N2—C13	118.19 (12)	N3—C12—H12A	109.2
C10—N2—C13	108.27 (11)	C13—C12—H12A	109.2
C14—N3—C12	112.69 (12)	N3—C12—H12B	109.2
C14—N3—C11	108.66 (12)	C13—C12—H12B	109.2
C12—N3—C11	110.14 (11)	H12A—C12—H12B	107.9
C14—N3—H3N	109.2 (11)	N2—C13—C12	109.53 (12)
C12—N3—H3N	108.5 (11)	N2—C13—H13A	109.8
C11—N3—H3N	107.6 (11)	C12—C13—H13A	109.8
C18—N4—C19	116.51 (14)	N2—C13—H13B	109.8
O1—C1—N1	122.03 (14)	C12—C13—H13B	109.8
O1—C1—C2	121.99 (13)	H13A—C13—H13B	108.2
N1—C1—C2	115.97 (14)	N3—C14—C15	115.00 (13)
C1—C2—C3	110.04 (14)	N3—C14—H14A	108.5
C1—C2—H2A	109.7	C15—C14—H14A	108.5
C3—C2—H2A	109.7	N3—C14—H14B	108.5
C1—C2—H2B	109.7	C15—C14—H14B	108.5
C3—C2—H2B	109.7	H14A—C14—H14B	107.5
H2A—C2—H2B	108.2	C16—C15—C19	117.83 (15)
C4—C3—C2	109.40 (13)	C16—C15—C14	122.94 (13)
C4—C3—H3A	109.8	C19—C15—C14	119.00 (15)
C2—C3—H3A	109.8	C15—C16—C17	120.04 (14)
C4—C3—H3B	109.8	C15—C16—H16	120.0
C2—C3—H3B	109.8	C17—C16—H16	120.0
H3A—C3—H3B	108.2	C18—C17—C16	116.57 (15)
C5—C4—C9	119.99 (15)	C18—C17—C20	121.22 (15)
C5—C4—C3	122.79 (15)	C16—C17—C20	122.21 (14)
C9—C4—C3	117.22 (14)	N4—C18—C17	125.19 (16)
C4—C5—C6	120.19 (16)	N4—C18—H18	117.4
C4—C5—H5	119.9	C17—C18—H18	117.4
C6—C5—H5	119.9	N4—C19—C15	123.81 (17)
C7—C6—C5	119.99 (15)	N4—C19—H19	118.1
C7—C6—H6	120.0	C15—C19—H19	118.1
C5—C6—H6	120.0	C25—C20—C21	118.47 (17)
C6—C7—C8	121.68 (14)	C25—C20—C17	120.38 (17)
C6—C7—H7	119.2	C21—C20—C17	121.13 (15)
C8—C7—H7	119.2	C22—C21—C20	120.67 (18)
C7—C8—N2	121.99 (13)	C22—C21—H21	119.7
C7—C8—C9	117.59 (14)	C20—C21—H21	119.7
N2—C8—C9	120.26 (13)	C21—C22—C23	120.2 (2)
C4—C9—N1	118.14 (13)	C21—C22—H22	119.9
C4—C9—C8	120.56 (14)	C23—C22—H22	119.9
N1—C9—C8	121.30 (13)	C24—C23—C22	119.25 (19)
N2—C10—C11	110.45 (12)	C24—C23—H23	120.4
N2—C10—H10A	109.6	C22—C23—H23	120.4
C11—C10—H10A	109.6	C25—C24—C23	120.72 (19)
N2—C10—H10B	109.6	C25—C24—H24	119.6
C11—C10—H10B	109.6	C23—C24—H24	119.6
H10A—C10—H10B	108.1	C24—C25—C20	120.67 (19)
C10—C11—N3	111.72 (13)	C24—C25—H25	119.7
C10—C11—H11A	109.3	C20—C25—H25	119.7
N3—C11—H11A	109.3		
			
C9—N1—C1—O1	−176.62 (13)	C14—N3—C12—C13	−172.97 (12)
C9—N1—C1—C2	2.4 (2)	C11—N3—C12—C13	−51.46 (16)
O1—C1—C2—C3	−143.54 (15)	C8—N2—C13—C12	165.68 (11)
N1—C1—C2—C3	37.46 (18)	C10—N2—C13—C12	−62.04 (14)
C1—C2—C3—C4	−56.18 (18)	N3—C12—C13—N2	57.34 (15)
C2—C3—C4—C5	−141.79 (17)	C12—N3—C14—C15	−69.10 (16)
C2—C3—C4—C9	38.3 (2)	C11—N3—C14—C15	168.56 (12)
C9—C4—C5—C6	0.5 (3)	N3—C14—C15—C16	95.52 (17)
C3—C4—C5—C6	−179.36 (17)	N3—C14—C15—C19	−90.00 (17)
C4—C5—C6—C7	−0.7 (3)	C19—C15—C16—C17	−1.5 (2)
C5—C6—C7—C8	0.8 (3)	C14—C15—C16—C17	173.04 (14)
C6—C7—C8—N2	174.79 (15)	C15—C16—C17—C18	0.1 (2)
C6—C7—C8—C9	−0.5 (2)	C15—C16—C17—C20	−179.93 (14)
C10—N2—C8—C7	−12.7 (2)	C19—N4—C18—C17	−1.9 (3)
C13—N2—C8—C7	116.78 (16)	C16—C17—C18—N4	1.7 (2)
C10—N2—C8—C9	162.54 (13)	C20—C17—C18—N4	−178.24 (15)
C13—N2—C8—C9	−68.01 (17)	C18—N4—C19—C15	0.4 (3)
C5—C4—C9—N1	−179.77 (15)	C16—C15—C19—N4	1.3 (3)
C3—C4—C9—N1	0.1 (2)	C14—C15—C19—N4	−173.47 (16)
C5—C4—C9—C8	−0.3 (2)	C18—C17—C20—C25	−39.3 (2)
C3—C4—C9—C8	179.60 (15)	C16—C17—C20—C25	140.79 (17)
C1—N1—C9—C4	−23.1 (2)	C18—C17—C20—C21	139.46 (17)
C1—N1—C9—C8	157.43 (14)	C16—C17—C20—C21	−40.5 (2)
C7—C8—C9—C4	0.3 (2)	C25—C20—C21—C22	−1.1 (2)
N2—C8—C9—C4	−175.13 (14)	C17—C20—C21—C22	−179.88 (15)
C7—C8—C9—N1	179.77 (14)	C20—C21—C22—C23	1.1 (3)
N2—C8—C9—N1	4.4 (2)	C21—C22—C23—C24	−0.4 (3)
C8—N2—C10—C11	−162.59 (12)	C22—C23—C24—C25	−0.3 (3)
C13—N2—C10—C11	63.20 (15)	C23—C24—C25—C20	0.2 (3)
N2—C10—C11—N3	−58.73 (16)	C21—C20—C25—C24	0.5 (3)
C14—N3—C11—C10	175.64 (11)	C17—C20—C25—C24	179.25 (15)
C12—N3—C11—C10	51.76 (15)		

### Hydrogen-bond geometry (Å, º)

*Cg* is the centroid of the C4–C9 ring.

**Table:**

*D*—H···*A*	*D*—H	H···*A*	*D*···*A*	*D*—H···*A*
N1—H1*N*···O1^i^	0.85 (2)	2.01 (2)	2.844 (2)	168 (2)
N3—H3*N*···Cl1^ii^	0.97 (2)	2.12 (2)	3.065 (1)	167 (2)
C10—H10*B*···O1^iii^	0.99	2.40	3.151 (2)	132
C11—H11*A*···Cl1^iii^	0.99	2.80	3.668 (2)	147
C12—H12*A*···Cl1	0.99	2.81	3.520 (2)	129
C12—H12*B*···O1^i^	0.99	2.26	3.123 (2)	144
C13—H13*A*···N1	0.99	2.53	3.138 (2)	120
C14—H14*A*···Cl1^iii^	0.99	2.71	3.585 (2)	147
C21—H21···Cl1	0.95	2.83	3.757 (2)	165
C18—H18···*Cg*^ii^	0.95	2.83	3.487 (2)	127

Symmetry codes: (i) −*x*, −*y*+1, −*z*; (ii) −*x*+1, −*y*+2, −*z*; (iii) *x*+1, *y*, *z*.

## References

[bb1] Feenstra, R. W., de Moes, J., Hofma, J. J., Kling, H., Kuipers, W., Long, S. K., Tulp, M. T. M., van der Heyden, J. A. M. & Kruse, C. G. (2001). *Bioorg. Med. Chem. Lett.* **11**, 2345–2349. 10.1016/s0960-894x(01)00425-5 11527728

[bb2] Feenstra, R. W., van den Hoogenband, A., Stroomer, C. N. J., van Stuivenberg, H. H., Tulp, M. T. M., Long, S. K., van der Heyden, J. A. M. & Kruse, C. G. (2006). *Chem. Pharm. Bull.* **54**, 1326–1330. 10.1248/cpb.54.1326 16946546

[bb3] Ghani, U., Ullah, N., Ali, S. A. & Al-Muallem, H. A. (2014). *Asian J. Chem.* **26**, 8258–8362.

[bb4] Groom, C. R., Bruno, I. J., Lightfoot, M. P. & Ward, S. C. (2016). *Acta Cryst.* B**72**, 171–179. 10.1107/S2052520616003954 PMC4822653 27048719

[bb5] Macrae, C. F., Sovago, I., Cottrell, S. J., Galek, P. T. A., McCabe, P., Pidcock, E., Platings, M., Shields, G. P., Stevens, J. S., Towler, M. & Wood, P. A. (2020). *J. Appl. Cryst.* **53**, 226–235. 10.1107/S1600576719014092 PMC6998782 32047413

[bb6] McKinnon, J. J., Jayatilaka, D. & Spackman, M. A. (2007). *Chem. Commun.* pp. 3814–3816. 10.1039/b704980c 18217656

[bb7] Sheldrick, G. M. (2008). *Acta Cryst.* A**64**, 112–122. 10.1107/S0108767307043930 18156677

[bb8] Sheldrick, G. M. (2015). *Acta Cryst.* C**71**, 3–8.

[bb9] Spackman, M. A. & Jayatilaka, D. (2009). *CrystEngComm*, **11**, 19–32.

[bb10] Spek, A. L. (2020). *Acta Cryst.* E**76**, 1–11. 10.1107/S2056989019016244 PMC6944088 31921444

[bb11] Stoe & Cie. (2009). *X-AREA* and *X-RED32*. Stoe & Cie GmbH, Darmstadt, Germany.

[bb12] Tan, S. L., Jotani, M. M. & Tiekink, E. R. T. (2019). *Acta Cryst.* E**75**, 308–318. 10.1107/S2056989019001129 PMC6399703 30867939

[bb13] Turner, M. J., McKinnon, J. J., Wolff, S. K., Grimwood, D. J., Spackman, P. R., Jayatilaka, D. & Spackman, M. A. (2017). *CrystalExplorer17.* University of Western Australia. http://hirshfeldsurface.net

[bb14] Ullah, N. (2012). *Z. Naturforsch. Teil B*, **67**, 75–84.

[bb15] Ullah, N. (2014*a*). *Med. Chem.* **10**, 484–496. 10.2174/15734064113096660046 24024527

[bb16] Ullah, N. (2014*b*). *J. Enzyme Inhib. Med. Chem.* **29**, 281–291. 10.3109/14756366.2013.776556 23488743

[bb17] Ullah, N. & Al-Shaheri, A. A. Q. (2012). *J. Chem. Sci.* **67**, 253–262.

[bb18] Ullah, N. & Altaf, M. (2014). *Crystallogr. Rep.* **59**, 1057–1062.

[bb19] Ullah, N., Altaf, M. & Mansha, M. (2017). *Z. Naturforsch. Teil B*, **58**, 1697–1702.

[bb20] Ullah, N., Altaf, M., Mansha, M. & Ba-Salem, A. O. (2015). *J. Struct. Chem.* **56**, 1441–1445.

[bb21] Ullah, N. & Stoeckli-Evans, H. (2014). *Acta Cryst.* E**70**, o103–o104. 10.1107/S160053681303448X PMC3998275 24764836

[bb22] Westrip, S. P. (2010). *J. Appl. Cryst.* **43**, 920–925.

