# Supplementary material for: Crystal structure and Hirshfeld surface analysis of the hydro­chloride salt of 8-{4-[(6-phenyl­pyridin-3-yl)meth­yl]piperazin-1-yl}-3,4-di­hydro­quinolin-2(1H)-one
Source: Acta Crystallogr E Crystallogr Commun. 2021 Jan 29;77(Pt 2):208–12. doi: 10.1107/S2056989021000979 (PMC7869540; doi:10.1107/S2056989021000979)
Supplement: Supplementary file 3 [file e-77-00208-sup3.pdf]

II.HCl-monohydrate

(Ullah & Altaf, 2014)   CCDC 987771 (CSD refcode AKUXEM)

Crystal data

$C_{25}H_{26}FN_4O^+\cdot Cl^-\cdot H_2O$	$F(000)$ = 496
M_r = 470.96	?
Triclinic, *P*	D_x = 1.348 Mg m^{-3}
Hall symbol: ?	Melting point: ? K
a = 8.3982 (8) Å	Mo *K*α radiation, λ = 0.71073 Å
b = 10.0514 (10) Å	Cell parameters from 10205 reflections
c = 13.9813 (14) Å	θ = 1.5–26.1°
α = 92.162 (8)°	μ = 0.20 mm^{-1}
β = 95.466 (8)°	*T* = 173 K
γ = 98.374 (7)°	Plate, colourless
V = 1160.7 (2) $Å^3$	0.45 × 0.32 × 0.15 mm
Z = 2	

Data collection

STOE IPDS 2 diffractometer	4393 independent reflections
Radiation source: fine-focus sealed tube	3097 reflections with $I > 2\sigma(I)$
Plane graphite monochromator	R_{int} = 0.080
Detector resolution: ? pixels mm^{-1}	θ_{max} = 25.7°, θ_{min} = 1.5°
φ + ω scans	*h* = −10 10
Absorption correction: multi-scan (*MULABS*; Spek, 2020)	*k* = −12 11
T_{min} = 0.549, T_{max} = 1.000	*l* = −16 17
13804 measured reflections	

Refinement

Refinement on F^2	Secondary atom site location: difference Fourier map
Least-squares matrix: full	Hydrogen site location: mixed

$R[F^2 > 2\sigma(F^2)]$ = 0.044	H atoms treated by a mixture of independent and constrained refinement
$wR(F^2)$ = 0.109	$w = 1/[\sigma^2(F_o^2) + (0.0655P)^2]$ where $P = (F_o^2 + 2F_c^2)/3$
S = 0.88	$(\Delta/\sigma)_{max}$ = 0.001
4393 reflections	$\Delta\rho_{max}$ = 0.33 e Å^{-3}
315 parameters	$\Delta\rho_{min}$ = −0.36 e Å^{-3}
0 restraints	Extinction correction: (*SHELXL2018*/3; Sheldrick, 2015), $Fc^*=kFc[1+0.001xFc^2\lambda^3/\sin(2\theta)]^{-1/4}$
? constraints	Extinction coefficient: 0.008 (2)
Primary atom site location: structure-invariant direct methods	

Supplementary Table S1. Hydrogen bonding (Å, °) in the crystal structure of **II.HCl** monohydrate

D—H···*A*	*D*—H	H···*A*	*D*···*A*	*D*—H···*A*
N1—H1*N*···O1*W*	0.84 (3)	2.07 (3)	2.853 (2)	156 (2)
N3—H3*N*···Cl1^{i}	0.89 (3)	2.20 (3)	3.0604 (17)	165 (2)
O1*W*—H1*WA*···Cl1	0.86 (3)	2.28 (3)	3.1264 (19)	166 (2)
O1*W*—H1*WB*···O1^{ii}	0.83 (3)	2.06 (3)	2.867 (2)	166 (3)
C10—H10*B*···O1^{iii}	0.99	2.55	3.328 (2)	135
C11—H11*B*···Cl1^{iv}	0.99	2.65	3.5164 (19)	146
C14—H14*A*···O1^{ii}	0.99	2.34	3.245 (2)	152
C14—H14*B*···Cl1^{iv}	0.99	2.79	3.633 (2)	143
C18-H18…Cg(C4-C9)^{v}	0.95	2.73	3.457(2)	134
Symmetry codes: (i) *x*, *y*+1, *z*; (ii) −*x*+2, −*y*+1, −*z*+2; (iii) *x*−1, *y*, *z*; (iv) −*x*+1, −*y*+1, −*z*+2 ; -x+2, -y+2, -z+2.				

Supplementary Figures, S1-S3, for structure **II.HCl** monohydrate (Ullah & Altaf, 2014).

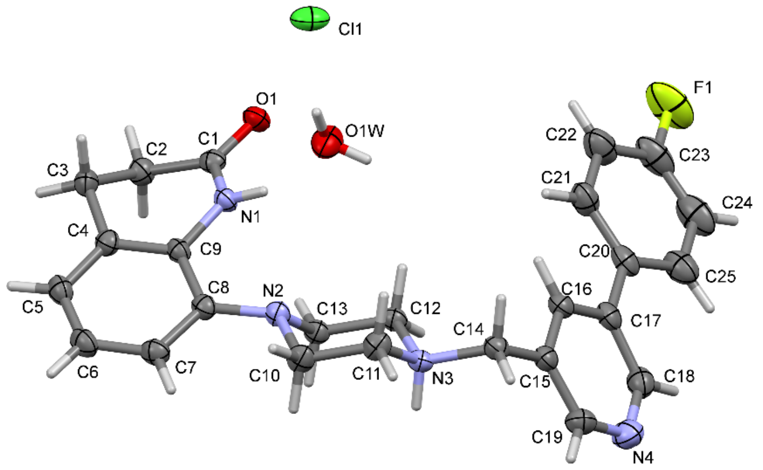

Fig. S1. Structure of **II.HCl** monohydrate, with atom labelling and displacement ellipsoids drawn at the 50% probability level.

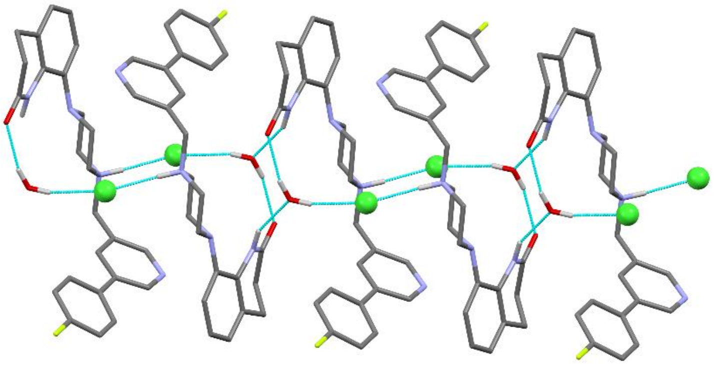

Fig. S2. A view of the hydrogen bonded chains of molecular salt **II.HCl** monohydrate, propagating alone the *b*-axis direction. The hydrogen bonds are shown as dashed lines and are tabulated above in Table S1.

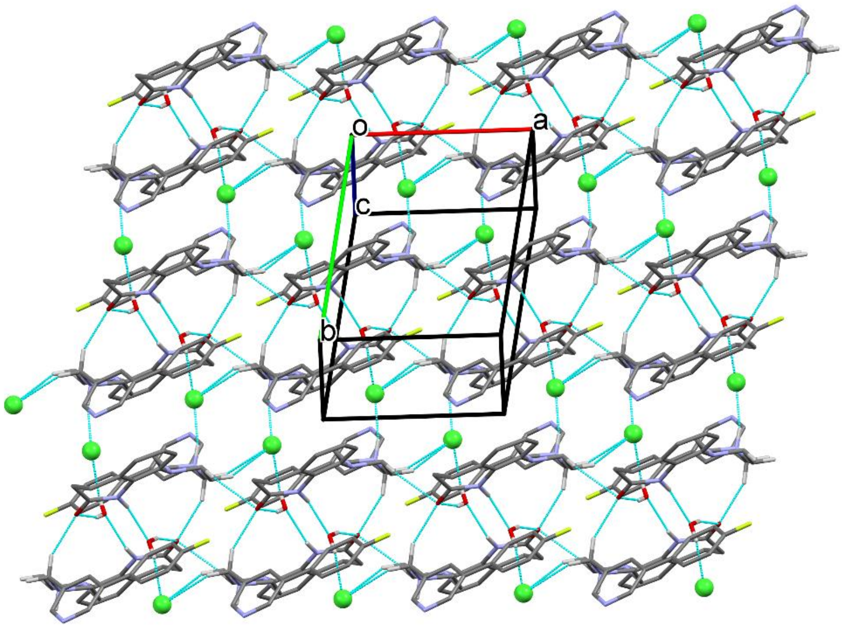

Fig. S3. A view of the layers formed by hydrogen bonding in the crystal of **II.HCl** monohydrate. The hydrogen bonds are shown as dashed lines and are tabulated above in Table S1.
